# Broadband enhancement of photoluminance from colloidal metal halide perovskite nanocrystals on plasmonic nanostructured surfaces

**DOI:** 10.1038/s41598-017-15230-x

**Published:** 2017-11-07

**Authors:** Si Zhang, Yuzhang Liang, Qiang Jing, Zhenda Lu, Yanqing Lu, Ting Xu

**Affiliations:** National Laboratory of Solid State Microstructures, College of Engineering and Applied Sciences, Collaborative Innovation Center of Advanced Microstructures, Nanjing University, Nanjing, 210093 China

## Abstract

Metal halide perovskite nanocrystals (NCs) as a new kind of promising optoelectronic material have attracted wide attention due to their high photoluminescence (PL) quantum yield, narrow emission linewidth and wideband color tunability. Since the PL intensity always has a direct influence on the performance of optoelectronic devices, it is of vital importance to improve the perovskite NCs’ fluorescence emission efficiency. Here, we synthesize three inorganic perovskite NCs and experimentally demonstrate a broadband fluorescence enhancement of perovskite NCs by exploiting plasmonic nanostructured surface consisting of nanogrooves array. The strong near-field optical localization associated with surface plasmon polariton-coupled emission effect generated by the nanogrooves array can significantly boost the absorption of perovskite NCs and tailor the fluorescence emissions. As a result, the PL intensities of perovskite NCs are broadband enhanced with a maximum factor higher than 8-fold achieved in experimental demonstration. Moreover, the high efficiency PL of perovskite NCs embedded in the polymer matrix layer on the top of plasmonic nanostructured surface can be maintained for more than three weeks. These results imply that plasmonic nanostructured surface is a good candidate to stably broadband enhance the PL intensity of perovskite NCs and further promote their potentials in the application of visible-light-emitting devices.

## Introduction

Colloidal metal halide perovskite nanocrystals (NCs) have recently attracted significant attention due to their outstanding optoelectronic characteristics. In particular, all-inorganic cesium halide perovskite (CsPbX_3_, X = Cl, Br, I) NCs exhibit surprisingly high photoluminescence (PL) quantum yield reaching 90% with narrow emission linewidths of 10~20 nm^1^. Compared with the traditional cadmium chalcogenide quantum dots, these CsPbX_3_ NCs have many unique advantages in PL properties, such as large tolerance to size and size distribution, low sensitivity to the surface dangling bonds, and easy color tuning over the entire visible spectral region via the ratios of mixed halide^2,3^. All these features make CsPbX_3_ NCs amazing emitting materials in the applications in LEDs^4,5^, lasing^6–8^, and photodetectors^9,10^.

As fluorescent materials have found extensive applications in optoelectronics and biological sciences, various techniques to improve the fluorescence efficiency and detection of the fluorescence signal have been widely studied in recent years^11–13^. One of the technique is to modify the emission with the presence of metallic nanostructured surface in close proximity to emitters^14^, mainly attributed to the generation of the surface plasmon polaritons (SPPs). SPPs are electromagnetic waves excited from the coupling of the electromagnetic fields and the oscillations of electron in the conductor, and propagate along the interface between conductor and dielectric^15^. Based on SPPs, many interesting physical phenomena and technologies have been explored and demonstrated, such as extraordinary optical transmission^16,17^, optical metamaterials^18–20^, optical negative refraction^21,22^, super-resolution imaging^23,24^ and surface enhanced Raman scattering (SERS) as well as plasmon-enhanced photovoltaics^25–28^.

The fascinating energy confinement characterization of SPPs in the perpendicular direction to the interface enables the plasmonic nanostructures to realize the enhancement of many optical effects including fluorescence emission^29,30^. The highly localized electromagnetic fields near the boundary can significantly boost the optical absorption of the fluorescent materials and result in an enhanced generation of electron-hole pairs. After energy relaxation within the fluorescent materials, the electron-hole pairs recombine and fluoresce. The radiation energy from fluorescent materials can be coupled with nanostructures again to excite the SPPs and directionally scattered into free space with high efficiency. In order to take the advantage of SPPs at the interface to achieve fluorescence enhancement, the excitation phase matching condition needs to be satisfied. One common method is using the surface array’s reciprocal vector to compensate the mismatch between the wavevector of SPPs and free-space photons. Previous researches have demonstrated the single wavelength fluorescence enhancement of semiconductor quantum dots via one- and two-dimensional plasmonic nanostructure arrays^13,31–34^.

In this paper, we synthesize all-inorganic CsPbX_3_ perovskite NCs with red, green and blue emitting colors, and study their fluorescence enhancement effect using plasmonic nanostructured surfaces. The plasmonic surfaces consisting of periodical metallic nanogrooves array can achieve broadband fluorescence enhancement across the visible spectral range, with a maximum enhancement factor reaching more than 8-fold for green light emitting NCs in experimental demonstration. In addition, as the CsPbX_3_ perovskite NCs are uniformly distributed in the polymer matrix upon the plasmonic nanostructures and isolated from air by cover layer in experiment, the fluorescence enhancement is very stable for the long-time operation. Our results indicate that, with a careful design, plasmonic nanostructures can be used to strongly enhance the fluorescence efficiency of perovskite NCs and promote their potentials in application of visible-light-emitting devices.

## Results and Discussions

**Figure 1:**
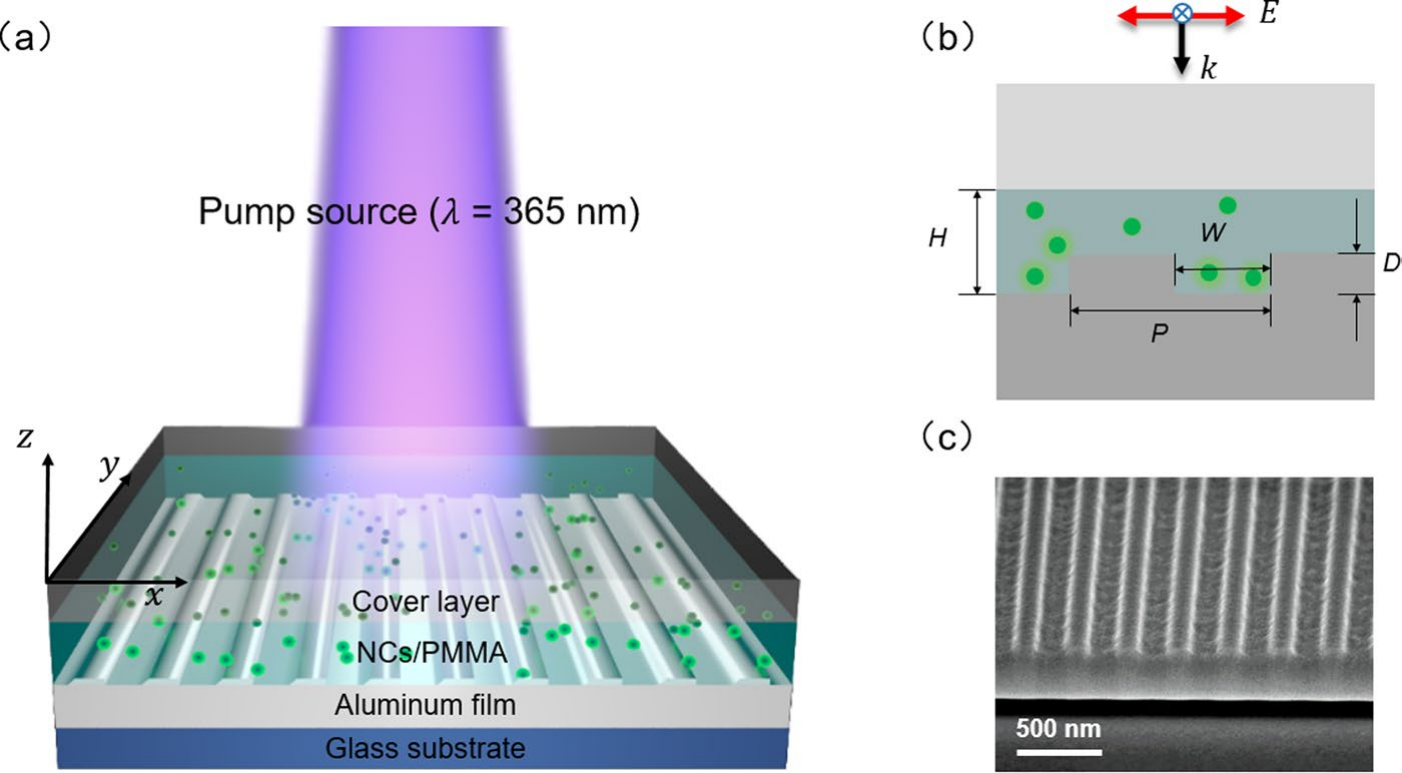
(**a**) Schematic of the structured surface with CsPbX_3_ perovskite NCs/PMMA composite film illuminated by an ultraviolet pump light. (**b**) The sketch of the cross section, where the electric field component vector is parallel to the array vector. (**c**) SEM image of the Al nanogrooves array (*P* = 300 nm, *W* = 150 nm).

Figure 1a shows the schematic diagram of the device where CsPbX_3_ NCs/polymethyl methacrylate (PMMA) composites are coating upon the plasmonic nanostructured surface. Here the plasmonic surface consists of periodical aluminum (Al) nanogrooves array, which is used to realize efficient conversion between free-space photons and SPPs. We use Al as a low-cost, earth-abundant metal that supports SPPs with low optical losses in the ultraviolet and visible regions of the spectrum^35,36^. Figure 1b illustrates the cross-section parallel to the periodical vector with feature sizes as following: nanogroove pitch *P*, width *W*, depth *D*, and NCs/PMMA layer thickness *H*. A scanning electron microscope (SEM) image of the fabricated Al nanogrooves array before spin-coating the NCs/PMMA solution is shown in Fig. 1c with *P* = 300 nm and *W* = 150 nm. Since SPPs only can be excited with transverse magnetic (TM) polarized light^15^, only TM polarized incidence (electric field component is perpendicular to the groove sidewall) is considered in this work.

**Figure 2:**
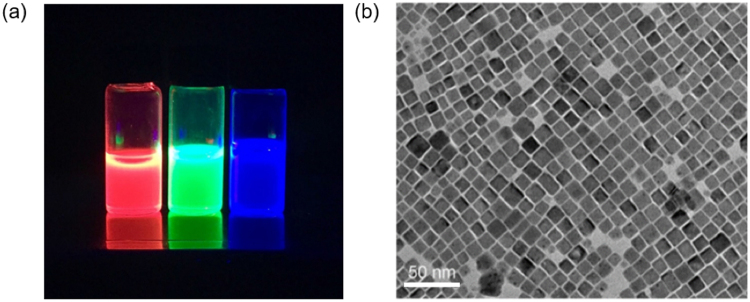
(**a**) CsPbX_3_ NCs and PMMA composite solution in toluene under excitation by an ultraviolet lamp (λ = 365 nm). (**b**) TEM image of the synthesized CsPbBr_3_ NCs.

Three different CsPbX_3_ NCs corresponding to red (R), green (G) and blue (B) emission colors are prepared by controlling the halide (Cl, Br, I) composition in the perovskite NCs. The main steps to obtain the CsPbX_3_ NCs/PMMA composite films include the nanocrystal synthesis and composite preparation. The details are given in the Methods Section. As shown in Fig. 2a, the fluorescence of the mixed solution of CsPbX_3_ NCs and PMMA excited by the ultraviolet light illumination clearly exhibit vivid RGB colors. Figure 2b shows a typical transmission electron microscopy (TEM) image of the synthesized CsPbBr_3_ NCs with a diameter about 10 nm, which is much smaller than the feature size of the Al nanogrooves. Therefore, it is possible to distribute the NCs into PMMA matrix uniformly and coat on the plasmonic nanostructured surface.

**Figure 3:**
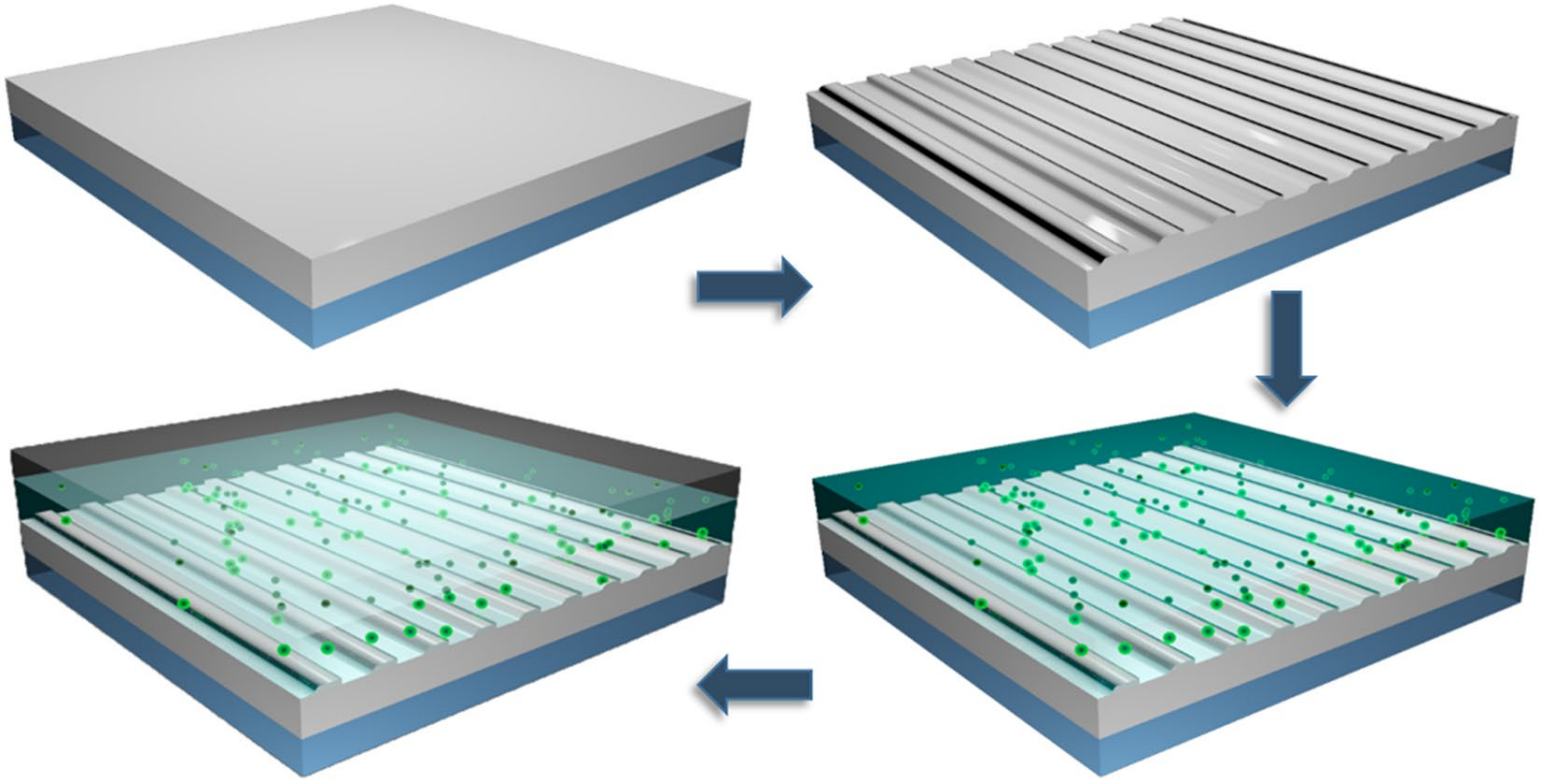
Illustration of fabrication process of samples. First, the Al film is deposited on the silica substrate via electronic beam evaporation. Then the nanogrooves array structure is defined by focus ion beam etching. After that, NCs/PMMA mixture is spin-coated on the structure. Finally, a layer of PMMA is coated on the top.

The fabrication procedure of the plasmonic nanostructured surface functionalized with NCs is shown in Fig. 3. Samples are processed on the standard quartz substrates that have been sputtered with a 280 nm-thick Al film. After Al deposition the nanogrooves array is patterned via focus ion beam etching (FIB, Helios NanoLab from FEI) with working beam restricted at 40 pA to control the groove slit width and depth. The total size of each metallic groove array is 20 µm × 20 µm, and the slit width and depth are fixed at 150 nm and 40 nm, respectively. Here, three fluorescence CsPbX_3_ NCs are studied: the red emitting CsPb (Br_0.2_I_0.8_)_3_ NC, the green emitting CsPbBr_3_ NC, and the blue emitting CsPb (Cl_0.5_Br_0.5_)_3_ NC. The composite mixtures of CsPbX_3_ NCs and PMMA solutions are spin-coated on the plasmonic nanostructured surface at a rotation speed of 3000 rpm, making the film thickness of the NCs/PMMA be approximately 200 nm. Then the sample is dried at 90 °C for 20 min. To preserve the activity of the CsPbX_3_ NCs, a protector layer is covered afterwards by spin coating another 200 nm-thick PMMA photoresist layer on the top, which is dried at 110 °C for another 20 min.

To study the enhancement effect of PL intensity in the entire visible spectral range systematically, three groups of samples coated with red, green and blue emitting perovskite NCs are prepared. Each group contains the four structures with nanogroove pitches ranging from 200 nm to 500 nm, fixed with slit width of 150 nm and depth of 40 nm. The PL intensities from perovskite NCs are experimentally measured using a UV-VIS-NIR microspectrometer (PV20/30 from CRAIC Technologies). The excitation light source is from a xenon lamp filtered with a laser filter at the wavelength of 365 nm, and a linear polarizer is introduced to select the TM polarized component of the incident light. In the reflective optical system, both the excitation light and the NCs’ PL are respectively focused and collected by a 20× objective lens with a numerical aperture (NA) of 0.45.

**Figure 4:**
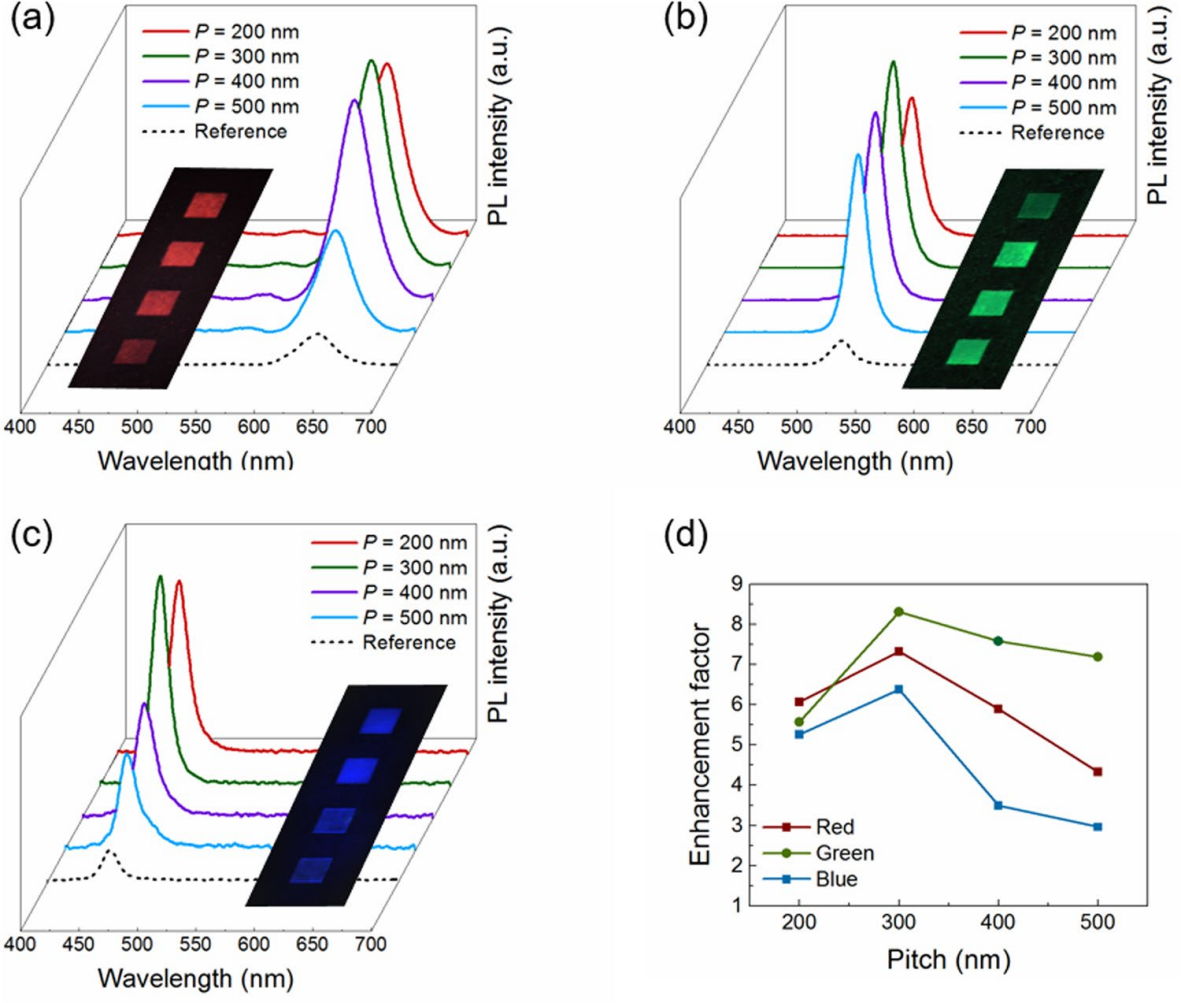
PL spectra and corresponding CCD images for (**a**) red, (**b**) green, and (**c**) blue emitting NCs/PMMA composite on the structures with different nanogrooves pitches. The reference group is taken from sample on the unpatterned metallic film. (**d**) Intensity enhancement factor of the PL spectra normalized to the measured peak intensity of NCs/PMMA on unpatterned Al surfaces.

The measured PL spectra from three groups are shown in Fig. 4a–c, where the PL spectra at unpatterned flat surface are also collected as the reference. It can be clearly seen that there is a remarkably multifold enhancement of the NCs’ PL intensity for all of three groups with the presence of the plasmonic nanogrooves array compared to the reference one. The enhancement factor is defined as normalizing the peak values of the PL intensity to that of the NCs/PMMA composite spin-coated directly on the unpatterned metallic surface, as shown in Fig. 4d. The maximum enhancement factors of the red, green and blue fluorescence signals reach about 7.3, 8.3 and 6.4, respectively. Besides the experimentally measured PL intensities, the fluorescence enhancement effect from the plasmonic nanostructured surfaces can also be easily obtained by the optical images taken by the fluorescence microscope. As we can see from the inset of Fig. 4a–c, these 20 µm × 20 µm blocks with nanogrooves array exhibit much stronger fluorescence signals than those of the surrounding unpatterned surfaces by the same optical excitation condition. In spite of distinctions in the maximum enhancement factors for different compositions of perovskite NCs, the plasmonic nanostructured surfaces remarkably achieve the broadband fluorescence enhancement in the entire visible range.

**Figure 5:**
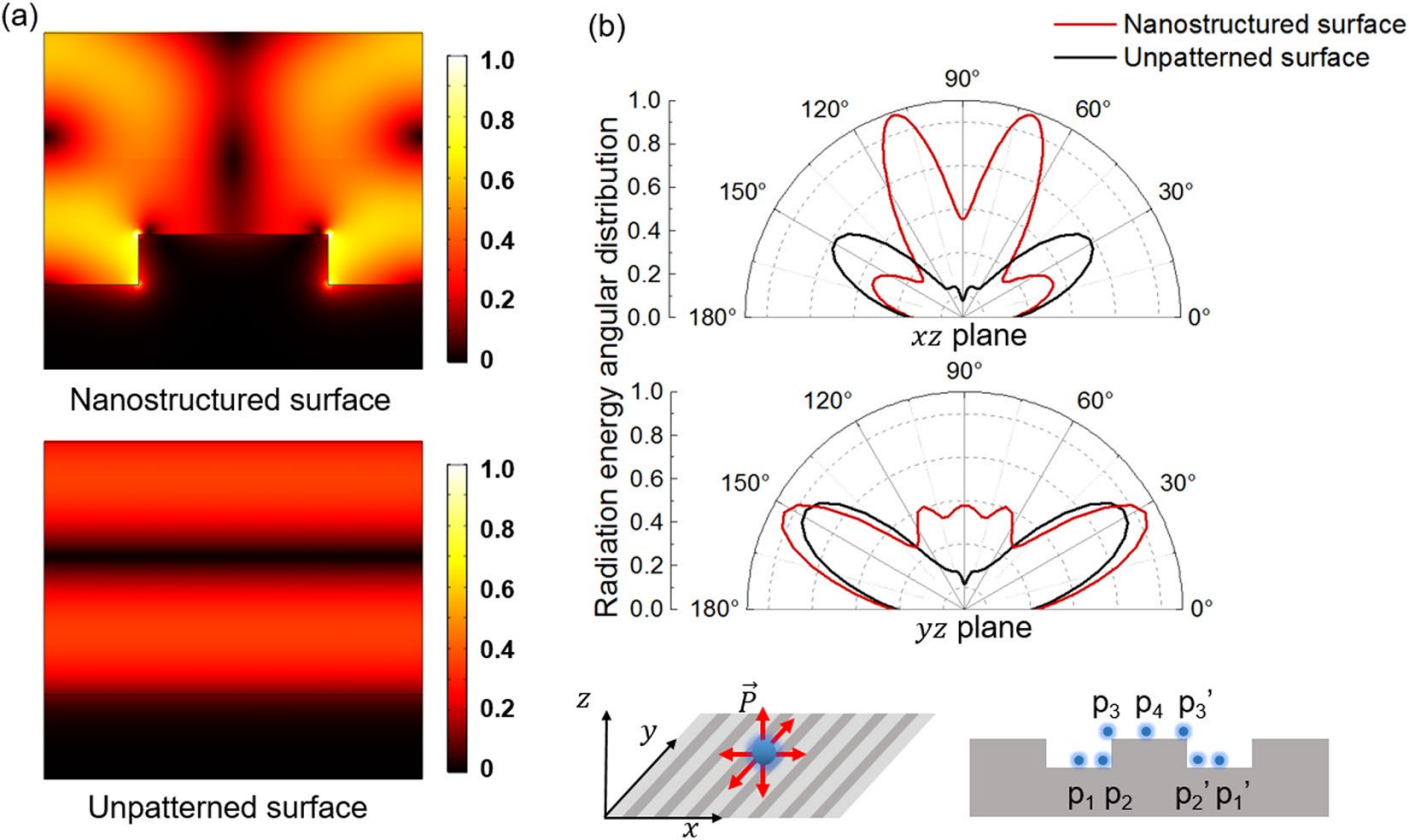
(**a**) Simulated normalized electric field distribution of nanostructured surface (top) and unpatterned (bottom) under TM polarized incident light at the wavelength of 365 nm. (**b**) Simulated far-field angular distribution of the radiation energy of an electric dipole with emission wavelength of 450 nm deposited on nanostructured (red line) and unpatterned surface (black line) in *xz* plane (top) and *yz* plane (bottom). The insets show the considered momentum directions and relative locations to the structure of the dipole.

In order to explain the fluorescence enhancement mechanism in detail, we numerically investigate the optical behaviors of the plasmonic nanostructured surface functionalized with NCs based on finite-difference time-domain (FDTD) simulations (Lumerical FDTD). We model the Al nanogrooves array with pitch *P* = 300 nm, slit width *w* = 150 nm and depth *h* = 40 nm. First, to study the procedure of optical excitation, we consider the excitation light as a normal incident plane wave at the wavelength of 365 nm, where the wavevector *k* is perpendicular to the plasmonic surface. As shown in Fig. 5a, compared with the referential unpatterned surface, there is a strongly enhanced local electric field near the metallic nanostructure under TM-polarized illumination. We integrate the norm of the electric field intensity in the dielectric layer on nanostructures, and get 2.5 times enhancement compared to the control sample. This originates from the excitation of SPPs where the nanogrooves array’s reciprocal vector compensates the phase mismatch between free-space photons and SPPs and realize the efficient conversion between them^15^. These strongly localized optical fields enhance the absorption of the perovskite NCs deposited close to the interface, thus leading to the PL intensity enhancement. In addition, the experiment results in Fig. 4d shows that the highest photoluminance enhancement always is achieved for the array with 300 nm pitch for all the three kinds of emitting nanocrystals. This is because the efficiency of excitation light transforming to SPP mode of structure surface is the highest for the array with 300 nm pitch leading to strong optical filed localization on the structure surface.

Besides the optical excitation, we also numerically investigate the PL emission procedure from the nanostructured surface in air. Figure 5c shows the calculated far-field angular distribution of the electric field intensities from a dipole emitter placed close to the nanostructured (red line) and unpatterned (black line) Al surfaces at the emitting wavelength of 450 nm. Here the dipole emitter contains three different electric polarization directions along *x*, *y* and *z*-axis and we average the electric field intensities from three different polarization directions. It can be clearly seen that at the *xz* plane, compared with unpatterned surface, the far-field PL intensity from nanostructured surface has a higher magnitude and a narrower divergence angle. This comes from the fact that the near-field evanescent PL emission from dipole emitter with high spatial frequencies can be coupled into SPPs at the metal-dielectric interface and then efficiently scattered by the nanostructured surface and reradiate into free space^37^. In addition, the plasmon-induced beaming light effect from the nanostructured surface also contributes to collimate the PL emission^38^, which benefits the PL signal collection by the objective lens with limited NA. The objective lens used in our experiments has a NA of 0.45, corresponding to a collection angle around ±26°. Therefore, in contrast to the unpattern surface, most of the PL emission from the nanostructured surface can be efficiently collected, which also contributes to the PL intensity enhancement. By integrating the emission energy distributed in the collection angle, the average enhancement factor of dipoles for *xz* and *yz* planes are estimated to be 5.9 times and 2.7 times enhancement, respectively. Here, the average enhancement factor is obtained through diploe source located on seven different positions of structure surface marked in the insets of Fig. 5(b), to avoid the influence of non-uniform distribution of electric filed on the structure grating on fluorescence enhancement. On the contrast, at the *yz* plane, the far-field PL emission from nanostructured surface has a more similar radiation angular distribution as the unpatterned surface, which is attributed to the absence of SPPs. Considering the two enhancement mechanisms above, the total enhancement factor is estimated around 10.7, a little higher than the experimental 6.4. This is because all nanocrystals in the theoretical calculation is placed on array surface with strongest localized optical filed. However, the nanocrystals in experiment are dispersed uniformly in the 200 nm-thick PMMA on the top of array surface. To summarize, compared with unpatterned surface, the PL intensity of perovskite NCs from nanostructured surface can be enhanced via increasing the optical absorption of pumping light (SPPs-induced near-field localization effect) and tailoring the PL emission (SPPs-coupled emission and beaming light effect).

**Figure 6:**
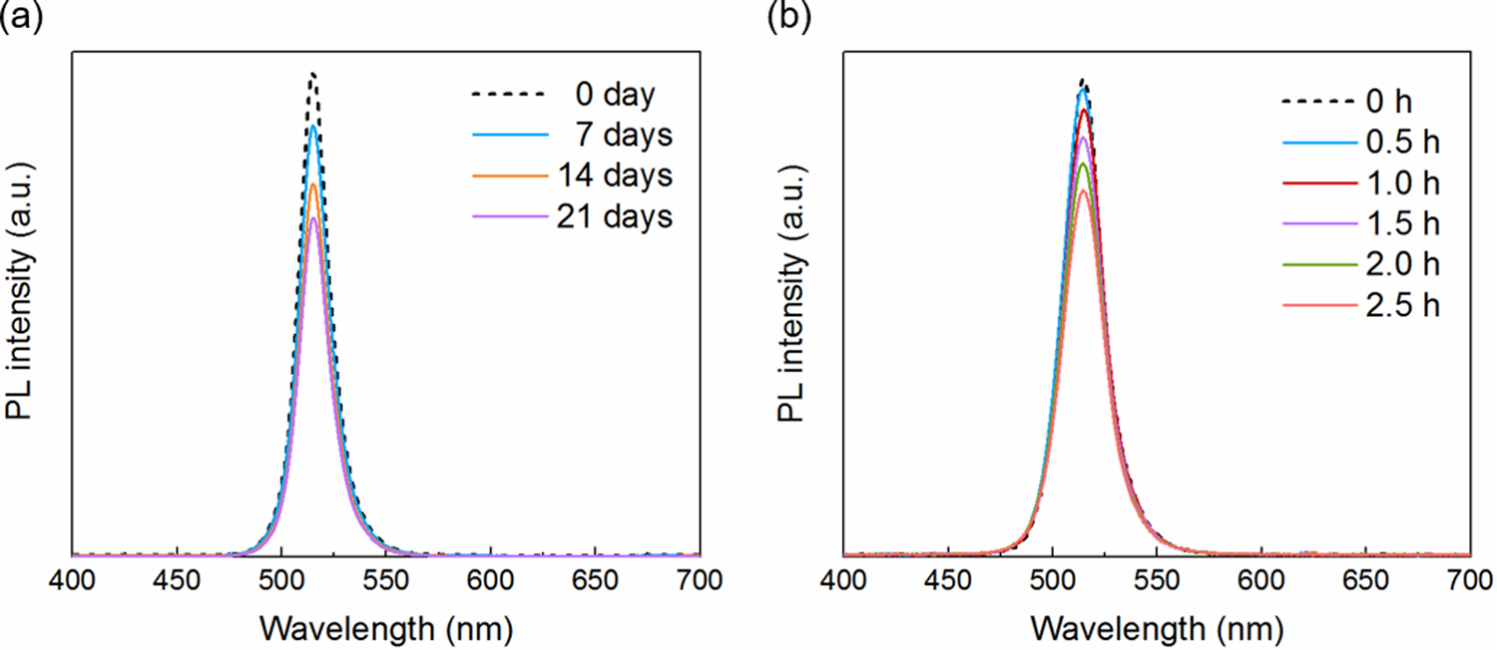
PL intensity spectra of the (**a**) protected and (**b**) unprotected plasmonic nanostructured surface coated with green NCs/PMMA with different storing times under ambient condition.

Operation stability is another very important factor for the perovskite NC-based light-emitting devices. Though the all-inorganic CsPbX_3_ NCs are much better than their organic-inorganic (methylammonium or other organic cation-based) counterpart, they are still quite unstable and would lose activity in hours exposed in atmosphere compared to other fluorescent dye and metal chalcogenide-based quantum dots. The extremely instable property of perovskite NCs has been a barrier for practical applications. Here, we spin-coat a 200-nm thick PMMA on the top of the NCs/PMMA composite acting as a protect layer, which significantly improve the PL stability of CsPbX_3_ NCs. Figure 6a shows the PL intensity of CsPbBr_3_ NC as a function of time. It turns out that the enhanced fluorescence signal is relatively stable, with an average of 4% intensity attenuation per week. After three weeks’ storage under ambient conditions, the sample still maintains high PL intensity. On the contrast, compared to samples without protector layer of which the signal decays rapidly as 1% intensity attenuation per hour in Fig. 6b. This stability enhancement should be highly encouraging from the perspective of the fluorescence signal enhancement in practical devices.

## Conclusions

In summary, we synthesize CsPbX_3_ NCs with RGB fluorescence colors and experimentally achieve multifold enhancement of PL intensity of NCs using plasmonic nanostructured surface consisting of Al nanogrooves array. The designed nanostructures can realize PL intensity enhancement for all three RGB fluorescence colors with a maximum enhancement factor of eight. We theoretically investigate the PL enhancement mechanism, including near-field optical absorption enhancement and SPP-coupled fluorescence emission. Moreover, with the presence of polymer protector layer in the device’s architecture, the fluorescence enhancement effect is very stable for the long-time operation. Therefore, the plasmonic nanostructured surface provides a good platform for the realization of high efficiency and stable perovskite nanocrystals-based visible-light-emitting devices.

## Methods

### Nanocrystal synthesis and composite preparation

CsPbX_3_ NCs are synthesized by the hot injection method according to the previous report^1^. Cs_2_CO_3_ (0.407 g) is loaded into a 50 mL three-neck flask along with octadecene (ODE, 20 mL) and oleic acid (1.25 mL). The mixture is degassed for 0.5 h at 120 °C, and then heated to 150 °C under N_2_ to form a clear solution. In another flask, ODE (5 mL) and PbX_2_ (0.188 mmol) are loaded along with oleylamine (1 mL) and oleic acid (0.5 mL). In our experiment, pure PbBr_2_ are added for green NCs emitters, while PbBr_2_/PbI_2_ (molar ratio 1:4) and PbBr_2_/PbCl_2_ (molar ratio 1:1) are for red and green ones, respectively. The mixture is then heated to 120 °C under vacuum, followed by a nitrogen gas flow when the temperature reaches 120 °C. After complete solubilisation of a PbBr_2_ salt, the temperature is raised to 150 °C, and followed by a quick injection of Cs-oleate solution (0.5 mL, prepared as described above). The reaction is stopped after 5 seconds by an ice-water bath. After the dispersion of perovskite NCs was cooled down to 50 °C, acetone with volume ratio 1:1 to original dispersion is added to precipitate perovskite NCs. The resulting NCs are collected by centrifugation, and then re-dispersed into 10 mL toluene. Meanwhile, the 6% wt PMMA is dissolved in toluene by magnetic stirring at 60 °C for 24 h. The CsPbX_3_ NCs colloidal dispersion prepared above is then mixed with equal volume PMMA solution, ultrasonicated for more than 30 min, followed by still standing for 20 min to prevent bubbles existing. After that, the CsPbX_3_ NCs/PMMA toluene dispersion is ready for spin coating on the fabricated Al substrate.

### Finite-difference time-domain numerical simulations

Finite-difference time-domain (FDTD) simulations (Lumerical FDTD) are performed to analysis the enhancement mechanism in absorption and emission part. The structure geometry consists of periodical nanogrooves array with a pitch of 300 nm, slit width of 150 nm and groove depth of 40 nm. The structured surface is embedded in NCs/PMMA composite with a thickness of 200 nm. The refractive indices of Al and PMMA is taken from refs^39,40^, respectively. To study the absorption enhancement, the composite system is illuminated by a normalized-incident plane wave source with wavelength of 365 nm and electric field component perpendicular to the groove sidewall. Periodical boundary condition is used to in the simulations. In the emission enhancement calculation, the NC is modeled as a single electric dipole with emission wavelength of 450 nm (correspond to the blue emitting NCs) on the nanostructured surface in air. Three dipole momentum directions along *x*, *y* and *z*- axis as well as seven different relative positions to the nanostructure as in Fig. 5b are considered in full 3D simulations. Ten periods of nanogroove are included in simulation area and perfect match layer boundary condition is applied. The far-field radiation energy angular distribution is obtained via the far-field analysis package.
